# Move for Change Part I: a European survey evaluating the impact of the EPDA Charter for People with Parkinson’s disease

**DOI:** 10.1111/j.1468-1331.2011.03532.x

**Published:** 2012-03

**Authors:** BR Bloem, F Stocchi

**Affiliations:** a Parkinson Centre Nijmegen, Donders Institute for Brain, Cognition and Behaviour, Radboud University Nijmegen Medical CentreNijmegen, The Netherlands; b Department of Neurology, Institute for Research and Medical CareIRCCS San Raffaele, Rome, Italy

**Keywords:** Charter, diagnosis, EPDA, European survey, guidelines, Parkinson’s disease, quality of care, treatment

## Abstract

**Background and purpose:**

The 1997 European Parkinson’s Disease Association’s (EPDA) *Charter for People with Parkinson’s disease* (PD) outlines their rights in terms of standards of care. It states that all patients have the right to: be referred to a doctor with a special interest in PD; receive an accurate diagnosis; have access to support services; receive continuous care; and take part in managing their illness. Move for Change is a three-part series of pan-European patient surveys based on this Charter.

**Methods:**

This first survey, consisting of 23 questions, focusing on the initial two points of the Charter, was administered online through the EPDA and affiliated patient associations’ Web sites. Of 2149 forms received from 35 European countries, 2068 (96.2%) were analyzed, with the remainder excluded, mainly due to incomplete responses.

**Results:**

The majority of patients were diagnosed within 2 years from the onset of first symptoms (82.7%; range, <1 year to ≥5 years). In relation to diagnosis delivery, 45.3% of patients stated that it was ‘poor’ or ‘very poor’. During the 2 years following diagnosis, 43.8% of respondents had never seen a PD specialist. Care was usually overseen by generically active neurologists (92.5%) or family doctors (81.0%), with considerable overlap between the two.

**Conclusions:**

These data highlight challenges that patients with PD face during the period of diagnosis, despite introduction of the Charter. These findings can assist healthcare professionals and policy makers in improving the level of care for patients and their families across Europe, and we offer suggestions about how this can be achieved.

## Introduction

Parkinson’s disease (PD) is the second most common neurodegenerative disease [1]. It is characterized biochemically by loss of dopamine through destruction of the nerve cells [2] and pathologically by the presence of α-synuclein-positive Lewy bodies within the *substantia nigra* [3]. The risk of PD increases with age [4]. It is estimated that there are approximately 1.2 million people with PD across Europe [5]. Onset is typically between 55 and 60 years of age [6], although approximately 4–5% of patients are diagnosed with ‘young–onset’ PD [4,7,8] (typically defined as developing <40 years of age).

Clinically, PD is known best for the presence of ‘motor symptoms’, including tremor, rigidity, bradykinesia, and postural instability [9]. PD is also associated with a wide range of ‘non-motor’ symptoms, which can occur before motor symptoms become apparent [10]. These include cognitive dysfunction, depression, sleep disturbances, and bladder dysfunction [11]. These non-motor symptoms are often poorly recognized and inadequately treated [2]. The combination of motor and non-motor symptoms causes a significant burden on the quality of life (QoL) of both patients and their carers [12,13]. A challenge for professionals involved in the care for patients with PD is that every case is different; not all patients will experience the same symptoms or weight them equally [14]. Treatment must therefore always be individualized. In addition to the impact on quality of life, the economic consequences of PD across Europe are considerable [5]. These include direct costs of consultations, hospital admissions and investigations, and indirect costs such as early retirement, reduced working hours, and institutionalization [6,15–17].

Symptomatic treatment is constantly improving, but it remains impossible to prevent PD or to slow down disease progression. Treatment typically involves a wide variety of medical and allied health disciplines [18]. Moreover, patients are increasingly demanding to be an active member of the multidisciplinary team [19]. To improve disease management, the European Parkinson’s Disease Association’s (EPDA) *Charter for People with Parkinson’s Disease* was launched in 1997, in conjunction with the World Health Organization (WHO). This Charter outlines the rights of patients in terms of standards of care [20–22], stating that all patients have the right to: be referred to a doctor with a special interest in PD; receive an accurate diagnosis; have access to support services; receive continuous care; and take part in managing the illness.

To evaluate whether the goals and ambitions of the Charter have been achieved, three pan-European patient surveys were designed for execution between 2010 and 2012. The first survey, presented here, examines whether the first two elements of the Charter are currently being met across Europe.

## Methods

### The survey

The Move for Change survey was targeted at people residing in Europe. The survey stated that it should be completed by a patient with PD, or a family member, carer, or healthcare professional on the patient’s behalf, and that only one questionnaire should be completed per patient.

The survey was administered through the EPDA Web site and the Web sites of affiliated national PD patient associations. People with PD were encouraged to participate in the survey by local PD organizations, but the survey was not actively promoted other than through the inclusion of a Web site banner.

Under guidelines from the European Pharmaceutical Research Association [23] and the UK National Health Service [24], the survey did not require the approval of Clinical Research Ethics Committee or Independent Review Board. Drug therapy was not addressed, and no adverse event reports were received. No incentive of financial/material reward was offered for the completion of the survey. Participation was voluntary. No personal data were gathered, ensuring respondents’ confidentiality and anonymity.

The survey was launched on 12 April 2010 to coincide with the European Parkinson’s Action Day and closed on 29 October 2010. The questionnaire contained 23 questions covering demographics, time to diagnosis, method of receiving a diagnosis, information available at the time of diagnosis, experience of diagnosis, follow-up visits, and involvement with Parkinson’s organizations or support groups. To allow for variation in clinical practice across Europe, the questionnaire included five options for the type of healthcare provider giving the original diagnosis, including a ‘Doctor with a specialist interest in Parkinson’s’– reflecting the wording within the original EPDA Charter and referring to a doctor who had more detailed knowledge and experience of the condition than a general neurologist or hospital doctor. *The original questionnaire is included as an online supplemental file* (Data S1).

Local PD associations translated the questionnaire; responses were completed in local language. Open responses were translated into English for central analysis. A total of 2149 forms were received, out of which 2068 were analyzed. Of those rejected, three were duplicates and the remainder had an insufficient number of questions answered.

### Analysis techniques and assumptions

Analyzed forms came from patients in 35 countries across Europe. Where the question related to diagnosis or the period immediately following diagnosis, the results were analyzed by country of residence at the time of diagnosis. For the remaining questions, the data were analyzed by the respondents’ current country of residence.

Countries with **<**10 respondents (i.e. **<**0.5% of the total survey sample) were not analyzed individually, but the results from these respondents were included in regional and European**-**level analyses. A total of 25 countries were analyzed at the country level, with 10 countries being excluded from individual analysis. A total of six respondents indicated that at the time of diagnosis, they were residing outside Europe (two in Asia, two in the USA, and two in the ‘rest of the world’). The results from these respondents have been excluded from questions that are based on country of residence at the time of diagnosis. Finally, 17 respondents did not state their current country of residence. The data for these respondents have been included within the European-level analysis only.

## Results

### Demographics

Of the 2068 questionnaires analyzed, 55.3% were from men. The most common age-group was 60–69 years (36.9%), with an overall mean age of 62.2 years. Two completed questionnaires (0.1%) were from patients <30 years of age, and 106 (5.1%) were from patients aged ≥80 years. Of the patients in the survey, 0.8% were diagnosed with PD before 1980 and 39.1% were diagnosed from 2006 to 2010. In total, 69.4% of patients were diagnosed since 2000. Additionally, 11.6% of respondents were diagnosed with PD before 1996, and the mean number of years since diagnosis was 8.3 years. Table 1 shows the regional distribution and demographic data for respondents.

**Table 1:** Geographic region distribution of respondents

	Mean gender					
		
Global region	Male (%)	Female (%)	Mean age (years)	Mean years since diagnosis (years)	Number of forms analyzed	Percentage of total analyzed
Eastern Europe						
Bosnia and Herzegovina^a^, Bulgaria, Czech Republic, Georgia^a^, Hungary, Poland, Romania, Russia^a^, Slovakia^a^	59.0	41.0	63.3	8.8	244	11.8
Northern Europe						
Denmark, Finland, Iceland^a^, Ireland, Lithuania, Norway, Scotland^b^, Sweden, United Kingdom, Wales^b^	48.0	52.0	62.5	7.4	608	29.4
Southern Europe						
Croatia^a^, Cyprus^a^, Greece, Israel, Italy, Malta, Portugal^a^, Slovenia, Spain, Turkey^a^	60.0	40.0	58.9	8.5	490	23.7
Western Europe						
Austria, Belgium, France, Germany, Luxembourg, Monaco^a^, Netherlands, Switzerland	57.0	43.0	64.1	8.8	709	34.3
Not stated					17	0.8
Total	55.0	45.0	62.2	8.3	2068	100

a Included in regional analysis; national sample too small to analyze individually.

b Included in UK data.

### Time to diagnosis

**Figure 1:**
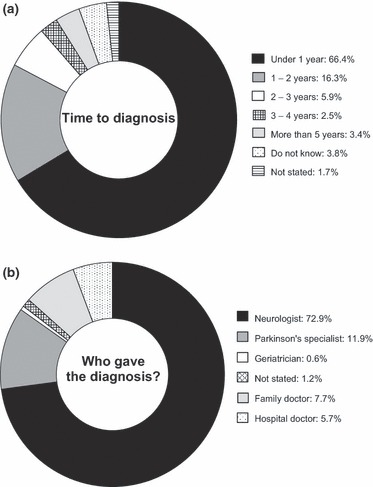
(a) Time to diagnosis after the onset of first symptoms (*n* = 2068). (b) Type of physician providing the diagnosis of PD (*n* = 2068).

The time to diagnosis, as estimated by patients, is shown in Fig. 1a. Two-thirds (66.4%) of patients were diagnosed within a year and 82.7% within 2 years. However, 11.8% took >2 years to receive a diagnosis of PD, and 3.4% waited >5 years.

Patients receiving a late diagnosis were most commonly middle-aged (50–69 years) and were more likely to have visited multiple specialists (more than three) to achieve this diagnosis. At the time of diagnosis, the majority of patients (70.7%) had seen either one or two doctors; in most cases, one of these was a neurologist. A third specialist physician was involved in 14.5% of cases. Western Europe had the highest proportion of people with PD diagnosed within 2 years (88.9%), whilst Southern Europe had the lowest (72.3%), suggesting a longer time to diagnosis.

### Method of receiving a diagnosis

The types of physicians giving the diagnoses are shown in Fig. 1b. Across Europe, the majority of patients (72.9%) received their diagnosis from a neurologist. A further 11.9% received the diagnosis from a doctor with a special interest in PD (PD specialist); these results are subject to respondent perception however, as there was no further definition of the level of specialist interest this may refer to. The percentage of patients receiving their diagnosis from a neurologist has risen steadily from 52.9% before 1980 to 76.7% in 2006–2010. Conversely, the percentage of PD specialists giving a diagnosis has dropped through the 1990s from a high of 19.0% in 1981–1985 to 9.9% in 2006–2010. The highest involvement of a PD specialist was seen in Eastern Europe (24.5%), with a similar involvement in Southern Europe (19.0%). In Northern Europe, 9.5% of diagnoses were carried out by a PD specialist; in Western Europe, this was just 4.6% of cases.

The majority (79.2%) of patients were aware of a PD specialist in their country, whilst 20% were not; 13.1% of whom did not know of a specialist.

**Figure 2:**
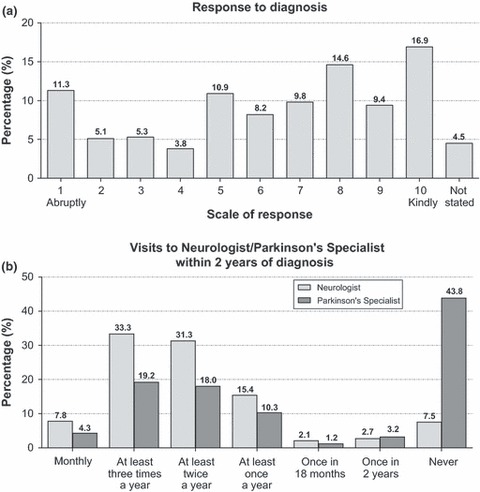
(a) Patients’ response to how their diagnosis of Parkinson’s Disease (PD) was given (*n* = 2068). (b) Frequency of visit to neurologist/PD specialist in the 2 years following diagnosis (*n* = 2068).

Almost all participants (96.9%) received their diagnosis in person; just 1.9% of patients in the survey received their diagnosis by telephone or letter, and only one individual by email. With regard to the delivery at the time of diagnosis, the majority of patients felt that they were dealt with in a kindly manner, with 58.9% giving a positive score (see Fig. 2a). The lowest scores, indicating ‘abrupt’ attitudes, were given most frequently when the diagnosis was handled by a neurologist or hospital doctor, and the highest scores, indicating ‘kindly’ attitudes, when dealing with a family doctor or general practitioner (GP).

### Information available at the time of diagnosis

A high proportion (62.2%) of patients reported receiving only general information following their diagnosis. Only 0.8% received no information at all, 22.1% reported receiving detailed information, and 14.0% reported receiving information on medication. Approximately 19.1% received information regarding a telephone helpline, but <2.8% received information on PD support organizations. Overall, the level of available information was greater in Northern and Western Europe, and lower in Southern and Eastern Europe. Amongst those who received information, 66.0% found the information given to be either ‘helpful’ or ‘very helpful’.

**Figure 3:**
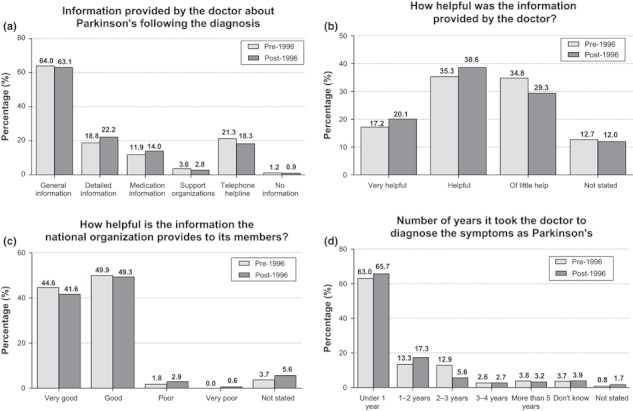
(a) Information provided by the doctor about Parkinson’s following a diagnosis pre-1996 (*n* = 241) and post-1996 (*n* = 1777). (b) Helpfulness of the information provided by the doctor following a diagnosis pre-1996 (*n* = 241) and post-1996 (*n* = 1777). (c) Helpfulness of the information provided by the national organization for participants with a diagnosis pre-1996 (*n* = 241) and post-1996 (*n* = 1777). (d) Number of years taken to diagnose the symptoms as Parkinson’s in participants who received a diagnosis pre-1996 (*n* = 241) and post-1996 (*n* = 1777).

Interestingly, there was little difference seen between those who were diagnosed before and after 1996 in terms of the level of information received. Approximately 64.0% received general information from their doctor when diagnosed pre-1996, compared with 63.1% of those diagnosed post-1996. Slightly more participants diagnosed post-1996 reported greater satisfaction with the information received, with 20.1% claiming it was ‘very helpful’ compared with 17.2% of those diagnosed pre-1996 (Fig. 3a–d).

### Experience of diagnosis

People with PD were asked to rate how they felt about the way in which their diagnosis of PD was delivered. Opinions varied, with 52.5% reporting that it was ‘good’ or ‘very good’, whilst 45.3% said ‘poor’ or ‘very poor’ (2.2% did not answer).

### Follow-up visits

Of those answering, almost half (43.8%) of the patients had never seen a PD specialist during the 2 years after being diagnosed with the disease (Fig. 2b).The majority of patients reported contact with a family doctor or neurologist, with 21% stating that they saw a family doctor at least three times a year (in connection with the PD diagnosis), and 33.3% reporting that they saw a neurologist at least three times a year (Table 2).

**Table 2:** Frequency of consultation in 2 years following diagnosis (a) by speciality, (b) by region

	Monthly (%)	At least 3 times a year (%)	At least twice a year (%)	At least once a year (%)	Once in 18 months (%)	Once in 2 years (%)	Never (%)
(a)							
Family Doctor	16.8	25.1	16.9	15.3	1.8	4.2	19.9
Hospital Doctor	3.6	10.3	12.2	13.0	1.7	3.5	55.8
Neurologist	7.8	33.3	31.3	15.4	2.1	2.7	7.5
PD specialist	4.3	19.2	18.0	10.3	1.2	3.2	43.8
Geriatrician	1.0	1.7	2.2	2.0	0.2	1.5	91.4

	Western Europe (%)	Eastern Europe (%)	Northern Europe (%)	Southern Europe (%)
(b)				
Family Doctor	84.4	82.9	68.7	86.2
Hospital Doctor	40.0	48.3	35.8	58.5
Neurologist	97.3	87.1	91.8	88.6
PD specialist	44.3	69.7	45.6	76.2
Geriatrician	7.3	9.3	7.3	12.4

The general neurologist predominated as the specialist caring for PD cases across Europe. This responsibility was shared to a significant (but varied) extent with the family doctor. ‘Hospital doctors’ were reported as being involved in some countries, but never to a great extent. Specialists in elderly medicine were rarely cited as being visited by people with PD.

**Figure 4:**
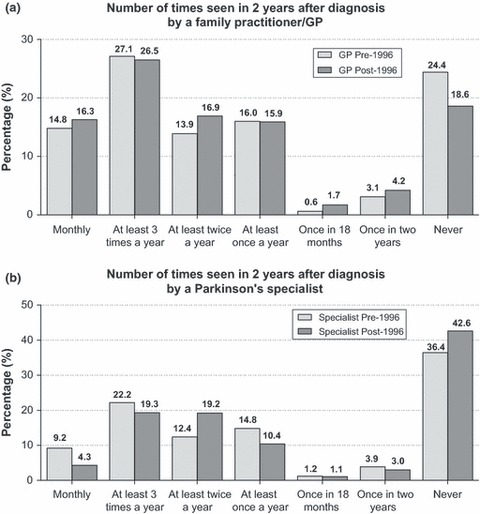
(a) Number of times a family practitioner/GP was seen in the 2 years following a diagnosis pre-1996 (*n* = 241) and post-1996 (*n* = 1777). (b) Number of times a family PD specialist was seen in the 2 years following a diagnosis pre-1996 (*n* = 241) and post-1996 (*n* = 1777).

When comparing data between participants diagnosed before and after 1996, the percentage of participants claiming never to have seen a family doctor or general neurologist in the 2 years following diagnosis has decreased from 24.4% pre-1996 to 18.6% post-1996. In contrast, the percentage of participants claiming never to have seen a PD specialist increased from 36.4% pre-1996 to 42.6% post-1996 (Fig. 4).

### Parkinson’s organizations and support groups

Amongst the respondents, 73.1% were members of a national PD organization, whilst 25.5% were not members, 1.5% did not answer, and 2.4% stated there was no organization in their country. Membership of a national organization was highest in Northern and Western Europe (85.7% and 88.6%, respectively).

Respondents who were not organization members were asked to provide reasons. Of the 287 reasons given, the most common (35.2%) was that they had no information on, or were unaware of, such organizations. A further 14.3% considered that it was too soon for them to join, either because their diagnosis was recent or because they felt no need for that type of support at present. Other reasons cited included the lack of local facilities, travel difficulties, expense, and time constraints owing to work. Of those who were a member of a national PD organization, 91.5% thought that the information provided by this organization was either ‘good’ or ‘very good’, and 73.1% found the services of these organizations ‘good’ or ‘very good’.

Approximately 64.8% of respondents reported that they did not use a support group, 26.8% used a local support group, 8.7% used an online support group, and 1.1% used a support group overseas.

## Discussion

Move for Change is the largest European patient survey on standards of care in PD to date. It was designed to identify any areas of care falling short of the standards within the 1997 EPDA Charter and current clinical guidelines [25–28]. Part 1 of the survey focuses on the diagnosis and referral to a PD specialist steps, and the results show considerable variation across Europe for these key actions. Because of the online format, it was not feasible to calculate a response rate in relation to the potential patient population for the European countries participating. However, consistent findings across the countries surveyed were that only a minority of patients were seen by a PD expert and that many patients with PD are dissatisfied with the way their diagnosis was conveyed.

The survey population is believed to be representative of the general European PD population. For example, amongst 802 patients with PD in Spain and Holland, the mean disease duration in three patient cohorts was 9.9, 11.0, and 7.7 years, respectively, and average ages were 60.8, 61.5, and 66.2 years [29]. Data for patients in the Move for Change survey are similar, with mean disease duration of 8.3 years and mean age of 62.2 years.

Although 82.6% of the survey participants received a diagnosis of PD within 2 years, approximately 11.9% were not diagnosed for more than 2 years. Current clinical guidelines recommend that once symptoms are suspected by a GP, the patient should be referred to a specialist for an accurate diagnosis within 6 weeks [26,30]. By the time a diagnosis was given, 14.5% of participating patients had seen three doctors, likely reflecting the variable non-motor symptoms that may present early or precede the motor phase of PD. It is unclear whether patients calculated their duration of disease from when they first noticed symptoms or the date of first reporting symptoms to a GP. Clarifying this in a future survey will help to identify whether there is a need for greater awareness of early symptoms amongst the general public, or whether more should be done to encourage those with symptoms to seek medical advice sooner.

Early referral to a specialist may reduce the period of uncertainty for patients awaiting a confirmed diagnosis and also reduce the rate of misdiagnosis. A study comparing the diagnostic accuracy of the clinical diagnosis of PD, as made by movement disorder experts or by non-expert physicians in the community, found a greater sensitivity (93.5% vs. 73.5%) and positive predictive value (88.7% vs. 73.5%) for the experts versus the non-experts. The negative predictive value was similar (76.9% for experts vs. 79.1% for non-experts) [31]. In our survey, only 11.9% of patients received their diagnosis from a PD specialist. As multiple non-conclusive evaluations and delays in diagnosis can be a drain on healthcare resources [32,33], streamlining the process of referral to a PD specialist and reorganizing the secondary care system [34] provide a cost-effective means of improving care for patients with PD.

The benefits of involving a specialist with expert understanding of PD may extend beyond the diagnostic steps [25,35]. For example, involvement of a movement disorder specialist results in greater adherence to key indicators of care quality in PD than when care is provided by a general neurologist [36]. Furthermore, a US study reported that patients seeing a PD specialist were up to three times more satisfied with their care than those seeing a general neurologist, possibly due to enhanced QoL [37]. Experiences in other neurologic conditions support the central role of specialists: stroke patients have better outcomes when treated on specialist stroke units rather than standard wards [38]; patients with multiple sclerosis are more likely to be prescribed innovative therapies by specialists than by general neurologists [39]. Additionally, patients who are given the opportunity to take an active role in their own disease management, for example by partaking in shared decision-making with their physician, have demonstrated better clinical outcomes, improved treatment adherence, greater QoL, and lower healthcare costs [40–43]. Future efforts should focus on further developing and implementing such patient-centered care for patients with PD.

Our study did not examine which factors currently limit access to PD specialists across Europe. However, barriers preventing access can include the need to travel to a specialist center, lack of funding, long waiting times, the need for a referral from a GP or a community neurologist, poor communication between a GP and specialist, lack of awareness amongst patients about the added value of a PD specialist, and inability to locate a PD specialist [44–46]. Further analyses within national and local healthcare systems are needed to identify which factors are most applicable and allow action plans to be developed to address these.

The experience of receiving their diagnosis significantly impacts on a patient’s health-related QoL [47]. However, almost half (45.3%) of the present survey respondents rated delivery of their diagnosis as ‘poor’ or ‘very poor’. Lower scores were given when the diagnosis was handled by a neurologist or a PD specialist. In contrast, family practitioners scored more highly, perhaps as they have more time or may know the patient on a more personal level. Our data suggest that, although neurologists are superior in terms of specialist knowledge, they should improve their communication skills. Communication skills training within the Continued Medical Education process could benefit general neurologists, PD specialists, and their patients.

Another concern identified here was the value of information provided to patients. Approximately 62.2% of the participants received general information following their diagnosis and, of those who responded, 34.0% felt the information was ‘of little help’. Additionally, <3% received any information on support organizations. As ‘satisfaction with the explanation of the condition at diagnosis’ is directly related to QoL [47], sufficient levels of appropriate information should be offered at this key point in patients’ lives. The pre-1996 and post-1996 diagnosis data suggest that the Charter has helped to improve this, but there is still room for further progress, for example by developing more tailored information materials providing essential information for patients, yet not overwhelming individuals coming to terms with their diagnosis.

This study has several limitations. First, this survey was only available online, thus restricting participation, particularly amongst older individuals. Additionally, individuals having Internet access often show an increased knowledge of their disease and available treatments and have higher expectations from physicians [48]. As such, the responses in this survey may be negatively biased in relation to the level of information received from the doctor. However, patients with Internet access are also most likely to seek access to the best care [48], so if anything, our inability to include patients without such access underscores the unmet needs in PD care in Europe.

Second, the absence of a universal definition for what constitutes a ‘Doctor with a specialist interest in Parkinson’s’ may have resulted in ambiguity for respondents. For example, a patient seeking good medical care may not always be aware whether their physician has undergone specialist training or has considerable clinical experience in PD management.

Third, up to 11.6% of the respondents were diagnosed with PD before 1996. Because these diagnoses took place before the introduction of the Charter, the issue of standards of care in PD management were yet to be raised and it was not explicitly advised for patients to receive an accurate diagnosis from a PD specialist, or adequate support services, *etc*. However, the opinions and experiences of these patients are still relevant for this survey, as they show the progression and development in PD management over time.

Fourth, some questionnaires could have been completed by individuals without connection to PD. In addition, due to the Web sites through which the survey was made available, those completing the survey may have had a higher awareness of the EPDA or a national PD association, and patients who are members of such organizations may be over-represented. This could have an upward effect on the approval ratings for information and support from these associations. These patients are also more likely to be actively involved in managing their disease, and be more aware of the options available to them. Again, this could lead to underestimation of the unmet needs identified by this survey.

A final limitation is the potential over- or under-representation of countries, as the distribution of respondents from each country within the European sample does not necessarily correlate with the national population. Any interpretations about international differences in care delivery should therefore be made cautiously.

Nonetheless, an online survey format provides several advantages: it can be completed at home at any convenient time; there is no need to arrange for transport to/from a clinical practice; and there is no burden for the healthcare professionals other than to direct their patients to the survey. In addition, online questionnaires achieve slightly higher completion rates than mailed questionnaires [49].

In conclusion, these results highlight certain challenges that people with PD still face surrounding the diagnosis of their condition. Despite the introduction of the Charter, few patients are referred to a specialist, the diagnosis can take more than 2 years and is often delivered in an unsatisfactory manner, particularly by a PD specialist, and the information patients receive is not well matched to their needs. We hope that these findings will assist in improving the level of care that is currently provided to patients and their families across Europe. We also encourage our colleagues and patients to participate in the next parts of this survey, thus providing more intelligence on how the care for patients can be improved.

## Supporting Information

**Data S1.** Original questionnaire used for Move for Change Part 1 survey
