# Supplementary material for: Move for Change Part I: a European survey evaluating the impact of the EPDA Charter for People with Parkinson’s disease
Source: Eur J Neurol. 2012 Mar;19(3):402–10. doi: 10.1111/j.1468-1331.2011.03532.x (PMC3489042; doi:10.1111/j.1468-1331.2011.03532.x)
Supplement: Supplementary file 1 [file ene0019-0402-SD1.doc]

**Online only: Original questionnaire used for Move for Change Part 1 survey**

Move for Change: European Action Day Survey 2010

1. Are you male or female? (**Please select one**)
   1. Male
   2. Female
2. What is your age? (**Please select one**)
   1. Under 30
   2. 30–39
   3. 40–49
   4. 50–59
   5. 60–69
   6. 70–79
   7. 80 and over
3. In what year were you diagnosed? (**yyyy**)
4. In which country do you live?
5. In which country were you living when you were diagnosed with Parkinson’s?
6. Who gave you the diagnosis of Parkinson’s? (**Please select one**)
   1. Family doctor or general practitioner
   2. Hospital doctor
   3. Neurologist
   4. Doctor with a special interest in Parkinson’s
   5. Physician specialising in care of the elderly/geriatrician
7. Do you know if your country has doctors with a special interest in Parkinson’s? (**Please select one**)
   1. Yes
   2. No
   3. I don’t know
8. How many doctors did you seek advice from about your Parkinson’s symptoms before the diagnosis was confirmed? (**Please select one**)
   1. 1
   2. 2
   3. 3
   4. More than 3
9. How long did it take for your doctor to diagnose your symptoms as Parkinson’s? (**Please select one**)
   1. Under 1 year
   2. 1–2 years
   3. 2–3 years
   4. 3–4 years
   5. More than 5 years
   6. I don’t know
10. How were you told by your doctor that you had Parkinson’s? (**Please indicate on a scale of 1 to 10**)

1 (Abruptly) 2 3 4 5 6 7 8 9 10 (Kindly)

1. How was your diagnosis given to you? (**Please select one**)
   1. In person
   2. Over the phone
   3. By letter
   4. By e-mail
2. Please describe your experience if it was very positive or very negative (**Blank space for verbatim response**)
3. What information did your doctor give to you about Parkinson’s following your diagnosis? (**Please tick all that apply**)
   1. General information about Parkinson’s
   2. Detailed information about Parkinson’s
   3. Support organisations
   4. Telephone help line
   5. No information
4. If the doctor provided information, how helpful was it to you? (**Please select one**)
   1. Very helpful
   2. Helpful
   3. Of little help

In the 2 years after your diagnosis, approximately how many times did you see the following healthcare professionals about your Parkinson’s?

1. Family doctor or general practitioner (**Please select one**)
   1. Monthly
   2. At least once a year
   3. At least twice a year
   4. At least 3 times a year
   5. Once in 18 months
   6. Once in 2 years
   7. Never
2. Hospital doctor (**Please select one**)
   1. Monthly
   2. At least once a year
   3. At least twice a year
   4. At least 3 times a year
   5. Once in 18 months
   6. Once in 2 years
   7. Never
3. Neurologist (**Please select one**)
   1. Monthly
   2. At least once a year
   3. At least twice a year
   4. At least 3 times a year
   5. Once in 18 months
   6. Once in 2 years
   7. Never
4. Doctor with a special interest in Parkinson’s (**Please select one**)
   1. Monthly
   2. At least once a year
   3. At least twice a year
   4. At least 3 times a year
   5. Once in 18 months
   6. Once in 2 years
   7. Never
5. Physician specialising in care of the elderly/geriatrician (**Please select one**)
   1. Monthly
   2. At least once a year
   3. At least twice a year
   4. At least 3 times a year
   5. Once in 18 months
   6. Once in 2 years
   7. Never
6. How do you feel your diagnosis was given? (**Please select one**)
   1. Very good
   2. Good
   3. Poor
   4. Very poor
7. Please describe your experience if it was very positive or very negative (**Blank space for verbatim response**)
8. Are you a member of a national Parkinson’s disease organisation? (**Please select one**)
   1. No
   2. Yes
      1. If not, why not? (**Blank space for verbatim response**)
   3. There is no patient organisation in this country
      1. If yes, how helpful is the information they provide? (**Please select one**)
         1. Very good
         2. Good
         3. Poor
         4. Very poor
      2. How beneficial are the services they provide? (**Please select one**)
         1. Very good
         2. Good
         3. Poor
         4. Very poor
9. Do you use a support group? (**Please tick all that apply**)
   1. No
   2. Local support group
   3. Online support group
   4. Overseas support group
